# Mechanical properties of gummies are associated with threat-related behavior and neuroglial markers in mice

**DOI:** 10.1016/j.jphyss.2026.100114

**Published:** 2026-10-23

**Authors:** Haruka Sasaki, Minori Tajima, Ririka Morimoto, Chihiro Kobayashi, Yumi Miki, Yuka Hirota, Hiromu Monai

**Affiliations:** a Department of Biology, Faculty of Science, Ochanomizu University, Bunkyo-Ku, Tokyo, 112-8610, Japan; b Institute of Human Life Sciences, Ochanomizu University, Bunkyo-Ku, Tokyo, 112-8610, Japan

**Keywords:** Gummy mechanical properties, Mastication, Food texture, Threat conditioning, Dentate gyrus, Microglia

## Abstract

Clenching is a sustained oral motor act distinct from rhythmic mastication, yet its contribution to brain function remains unclear because oral-function models often alter sensorimotor variables simultaneously. Because chewing under stress has been linked to hippocampal plasticity and the dentate gyrus is implicated in contextual fear conditioning, we compared mice given powdered chow with Soft or Hard gummies, with Powder and Pellet as references, and assessed basal behavior, object recognition, threat conditioning, and dentate gyrus DCX labeling, with cortical glial markers examined in parallel. Basal behaviors and between-group object recognition did not differ significantly, whereas contextual freezing was higher in Hard and Pellet than in Soft. Hard also showed higher dentate gyrus DCX immunoreactivity and cortical Iba-1 measures, while cortical GFAP was unchanged. These findings suggest that gummy mechanical properties are associated with threat-related behavior and neuroglial markers, motivating direct tests of oral motor load and neural mechanisms.

## Introduction

Clenching is an oral motor act within the masticatory system, but it is fundamentally distinct from rhythmic mastication, which serves to break down and transport a food bolus. Whereas mastication consists of rhythmic jaw opening, closing, and occlusion, clenching is a high-load oral motor act characterized by sustained, near-isometric force generation around the occlusal position. Indeed, jaw-muscle activation patterns and jaw gape differ between chewing and isometric biting, even when comparable levels of bite force are produced [1]. Voluntary teeth clenching has also been reported to increase isometric ankle plantar-flexion strength [2], facilitate the soleus H-reflex and reflex activity of pretibial muscles [3], [4], and enhance motor system excitability in both upper and lower limbs [5]. Thus, clenching should be regarded not merely as a component of feeding behavior but as an oral motor act associated with high-effort states and systemic motor facilitation.

In contrast, most studies examining the relationship between oral function and brain function have focused on the effects of reduced mastication. Soft-diet feeding after weaning reduces adult neurogenesis in the hippocampal dentate gyrus [6], and reduced mastication has been linked to memory impairment accompanied by morphological and activity-related changes in the hippocampus [7]. Conversely, stronger masticatory loading has been associated with increased survival of newborn cells in the dentate gyrus [8]. Chewing under restraint stress has also been reported to attenuate stress-induced suppression of cell birth in the dentate gyrus [9], ameliorate stress-induced impairment of hippocampus-dependent spatial memory [10], and suppress stress-induced hippocampal long-term depression and anxiety-like behaviors [11].

However, many of these experimental models rely on soft-diet feeding, tooth extraction, bite-raising, or chewing interventions under stress, which may alter different combinations of masticatory activity, oral sensory input, occlusal support, food texture, nutritional conditions, surgical injury, pain, and stress-buffering effects. A systematic review of animal studies also indicated that the major experimental models used to examine hippocampal morphology and function were soft-diet feeding, tooth extraction, and bite-raising [12]. Therefore, the specific contribution of clenching to brain function, independently of broader changes in mastication and oral sensory input, remains insufficiently resolved. A related limitation is also present in human observational studies: although occlusal force has been associated with cognitive function in community-dwelling older adults [13], such findings do not isolate the specific contribution of clenching load from broader oral function and general health status. Therefore, basic experimental models combining dietary manipulation with direct measurements of oral motor behavior are needed to dissect how this oral motor variable contributes to brain function.

To examine this issue, we selected behavioral tests and cellular markers that could plausibly reflect the effects of oral motor load. Previous studies of reduced mastication have reported impairments in memory and learning function [7], [12], raising the possibility that altered oral motor input may affect cognitive performance. Therefore, we first performed the novel object recognition test to compare performance in a basic non-emotional recognition-memory task between the Soft and Hard dietary conditions. We then performed threat-conditioning tests because chewing under stressful conditions has been reported to modulate hippocampal plasticity and anxiety-like behaviors [9], [10], [11], and because dentate gyrus function and adult hippocampal neurogenesis have been implicated in contextual fear conditioning and pattern separation [14], [15], [16]. Accordingly, dentate gyrus DCX labeling was examined as an immature neuron-related marker.

In parallel, cortical GFAP and Iba-1 labeling were examined as exploratory glia-related histological markers to assess whether differences in gummy mechanical properties are accompanied by differences in immunoreactivity outside the dentate gyrus. The rationale for examining cortical markers was that orofacial sensory information is conveyed through trigeminal pathways and represented in somatosensory cortical areas, and that the rodent oral somatosensory cortex has been implicated in oral sensory processing and food-consummatory orofacial movements [17], [18].

Based on this rationale, we established a dietary model using gummies with different mechanical properties. Mice were assigned to four dietary conditions: powdered chow alone (Powder), powdered chow supplemented with low-compression-load gummies (Soft), powdered chow supplemented with high-compression-load gummies (Hard), or standard pellet chow (Pellet). The Soft and Hard groups served as the principal comparison of gummy mechanical properties, while Powder and Pellet served as reference conditions. Using this model, we first assessed body weight and basal behaviors. We then tested whether the dietary gummy conditions were associated with non-emotional object recognition and threat-related behavioral indices, and whether these behavioral measures were accompanied by changes in dentate gyrus DCX labeling and cortical GFAP/Iba-1 labeling. Through this approach, we aimed to examine how dietary conditions differing in gummy mechanical properties are associated with behavioral and neuroglial measures relevant to oral-brain interactions.

## Materials and methods

### Animals and experimental design

All experimental procedures were approved by the Institutional Animal Care and Use Committee of Ochanomizu University, Japan (protocol nos. 23007, 24017, and 25007). All animal experiments were performed in accordance with the Guidelines for Animal Experimentation of Ochanomizu University, which conform to the Fundamental Guidelines for Proper Conduct of Animal Experiments and Related Activities in Academic Research Institutions of the Ministry of Education, Culture, Sports, Science and Technology, Japan. All efforts were made to minimize the number of animals used. Details of allocation and exclusions are reported below.

Wild-type C57BL/6 J mice were used in this study. Male mice were used for the main behavioral and histological cohorts; the separate LED cue-associated avoidance cohort included both sexes. For the male cohorts, mice were weaned at postnatal day 21 and assigned to one of four diet groups at postnatal day 28 (P28). They were maintained under the assigned diet conditions until P63 (Fig. 1). Each dietary group was housed in two cages. No formal randomization procedure was used. Mice were maintained under a 12-h light/dark cycle (lights on from 08:00–20:00) at 22–24 °C, with ad libitum access to food and water. The main behavioral assessments were conducted as a fixed-order test battery in the same mice. Test order was not counterbalanced. Exact sample sizes for each experiment are indicated in the figure legends. The individual mouse was treated as the unit for behavioral comparisons and the additional Iba-1 image analysis, for which one field per mouse was analyzed.

**Fig. 1:**
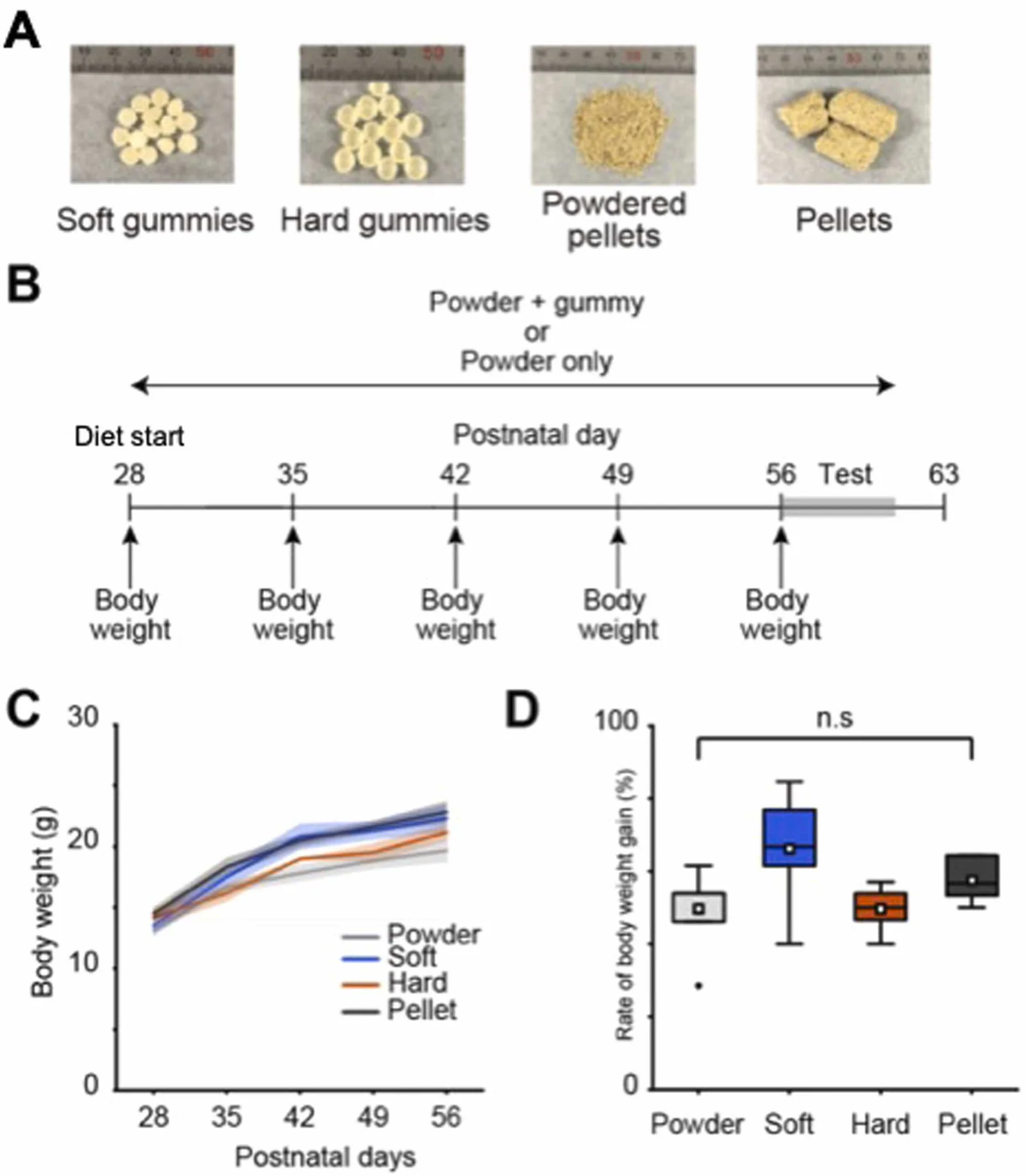
Experimental design and body weight under four diet conditions. (A) Representative images of the four diet conditions: Soft, Hard, Powder, and Pellet. (B) Experimental schedule. (C) Body weight from P28 to P56 in the four groups. (D) Percentage increase in body weight from P28 to P56. Numerical body-weight summaries in the text are mean ± SEM. n = 6 mice per group. The P28–P56 percentage increase was analyzed by one-way ANOVA followed by Tukey’s multiple-comparison test. The bracket in D indicates the Powder–Pellet comparison; n.s., not significant (Tukey-adjusted).

For the main four-group experiments, the target sample size was six mice per group, based on our previous experimental experience and the anticipated variability of the measurements. No a priori sample-size calculation or power analysis was performed. When feasible, a seventh mouse was tested and included in the analysis; this was decided before examining group comparisons or statistical test results. The behavioral analysis approach was developed using practice animals before the main experiments; their data were not included in any reported analyses.

### Diet and gummy conditions

Mice in the Powder group were fed powdered chow only. Mice in the Soft group were fed powdered chow supplemented with soft gummies, whereas mice in the Hard group were fed powdered chow supplemented with hard gummies. Mice in the Pellet group were fed standard pellet chow. Powdered chow was prepared by grinding the same standard chow pellets (PicoLab Rodent Diet 20, PMI Nutrition International) and passing the material through a 2-mm mesh sieve.

Two types of gummies with different mechanical properties were prepared for this study by the Food Science Institute Foundation (Ryoushoku Kenkyukai, Japan). To evaluate the mechanical differences between the two gummy types, the maximum load required to compress each gummy to a strain of 0.8 was measured using a compression-tension rheometer. The hard gummies contained 8.6% gelatin, had a maximum compression load of 80 N, and weighed 0.1 *g* per piece. The soft gummies contained 2.6% gelatin, had a maximum compression load of 50 N, and weighed 0.1 *g* per piece. Both gummy types contained 0.76 g/g sugar, and their energy content and nutritional composition were adjusted to be as similar as possible. Gummies were supplied at an amount equivalent to 7 *g* per mouse per week. The daily amount was divided equally and supplied into the cage using an automatic feeder. Mice in the Soft and Hard groups had free access to both powdered chow and gummies, whereas mice in the Pellet and Powder groups received pellet chow only or powdered chow only, respectively. These compression-load values characterize the mechanical properties of the gummies and do not represent bite force or clenching force exerted by the mice. Actual gummy consumption was not quantitatively recorded.

### Behavioral testing

Behavioral tests were conducted during the light phase. Behavior was recorded from above using a video camera and analyzed offline using MATLAB (MathWorks, USA), DeepLabCut [19], ImageJ, and custom scripts as required. The apparatus was cleaned with 70% ethanol between animals.

### Open field test

To assess spontaneous locomotor activity and anxiety-like behavior, mice in the Soft and Hard groups were subjected to the open field test. The test was performed in a square arena measuring 40 × 40 cm with 40-cm-high walls. Mice underwent a 5-min habituation session in the same arena before the OFT. The 5-min OFT was video-recorded at 25 frames/s. The entire 5-min recording was used for quantitative analysis. Locomotor trajectories were extracted using MATLAB, and total distance traveled (m) was calculated from successive centroid positions of the mouse region extracted by background subtraction, using a video-specific spatial calibration. A 150 × 150-pixel center zone, corresponding to approximately 13 × 13 cm, was positioned visually at the arena center. Center-zone time (s) was calculated by summing the mouse-region pixels overlapping this zone across frames, dividing by the mouse-region pixel count per frame and by the frame rate. No animals were excluded from this analysis.

### Tail suspension test

To assess depression-like behavior, mice were subjected to the tail suspension test. Each mouse was suspended by adhesive tape placed approximately 3 cm from the tail tip, and behavior was video-recorded for 10 min at 30 frames/s. The final 5 min were used for analysis. Immobility was scored visually in ImageJ, and the number of frames scored as immobile was divided by the frame rate to obtain immobility time. Behavioral scoring was performed without knowledge of group allocation. No animals were excluded from this analysis.

### Novel object recognition test (NOR)

To assess non-emotional object recognition memory, mice in the Soft and Hard groups were subjected to the novel object recognition test. The test was performed in a rectangular acrylic cage measuring 30 × 15.5 cm, under controlled illumination and environmental noise conditions. On P55, mice were habituated to the empty arena for 5 min. On P56, mice were first re-exposed to the empty arena for 5 min. Two identical spherical objects (Object A/A) were then placed symmetrically in the arena, and mice were allowed to explore them for 10 min during the familiarization phase. After an 8-min interval in the home cage, mice were re-exposed to the empty arena for 5 min. One of the familiar objects was then replaced with a triangular-pyramid object (Object B), and mice were allowed to explore the objects for 10 min during the test phase. Both phases were recorded at 30 frames/s, and the first 5 min of each 10-min recording were used for analysis.

Behavior was recorded from above. Exploration time was defined as the duration for which the mouse’s nose, tracked using DeepLabCut, was located within a circle of radius 3 cm centered on an object. Time spent on an object was included when the nose satisfied this spatial criterion. The discrimination index (DI) was calculated as follows:

DI = (*T*_B_ − *T*_A_) / (*T*_B_ + *T*_A_)

where TA indicates the exploration time for the familiar object (Object A) and TB indicates the exploration time for the novel object (Object B). A DI of zero represents equal exploration of the two objects. No minimum exploration-time exclusion criterion was prespecified. One mouse in the Soft group was excluded because it escaped from the arena during testing and the required data could not be obtained, resulting in final sample sizes of Soft n = 5 and Hard n = 7.

### Threat conditioning and behavioral assessment

Freezing in the conditioning chamber was assessed in the Powder, Soft, Hard, and Pellet groups, whereas LED cue-associated avoidance was assessed in a separate mixed-sex Soft–Hard cohort. The conditioning chamber was equipped with a metal grid floor (ENV-414S, MED Associates, USA). Both cohorts received the same conditioning protocol on five consecutive days from P56 to P60 (Monday to Friday), including a conditioning session before behavioral assessment on Friday (P60). Each daily session began with a 5-min habituation period and comprised five trials. In each trial, a green LED was illuminated for 20 s, and a 0.5-mA foot shock was delivered during the final 0.5 s of illumination. Consecutive trials were separated by a 90-s intertrial interval.

After the final conditioning trial on Friday, mice in the contextual-freezing cohort were returned to their home cages. At least 30 min after the final foot shock, mice were reintroduced into the conditioning chamber, and behavior was recorded for 5 min. No foot shocks were delivered, and the LED remained off throughout this observation period. Freezing was defined as the absence of body movement except for respiration. Freezing was scored visually in ImageJ without knowledge of dietary group allocation. The recording was divided into 30 consecutive 10-s intervals, and each interval was scored as freezing-positive if at least 2 s of freezing was observed within that interval. The freezing rate was calculated as the number of freezing-positive intervals divided by 30 and multiplied by 100. All six mice tested in each dietary group were included in the contextual-freezing analysis; no animals were excluded from this analysis.

The LED cue-associated avoidance test was also performed on Friday following that day’s conditioning session. A two-compartment apparatus was used in which only one compartment could be illuminated with the same green LED. Mice were placed in the apparatus for 5 min of habituation and were then allowed to freely explore the apparatus for 5 min. This test was performed in a separate mixed-sex cohort (Soft, n = 11: 6 females and 5 males; Hard, n = 12: 6 females and 6 males). Total time spent in the LED-on compartment was recorded for each mouse, and individual total durations were used for the Soft–Hard comparison. For descriptive visualization, the available individual visit durations (Soft, 69 visits; Hard, 82 visits) were pooled within each condition and displayed as count histograms using common 5-s bins spanning 0–80 s. The reported Soft–Hard comparison pooled sexes. The visit-duration histograms were not used for inferential group comparisons.

### Immunohistochemistry

After completion of the designated behavioral tests, mice were deeply anesthetized and transcardially perfused with 0.1 M phosphate-buffered saline (PBS), followed by 4% paraformaldehyde (PFA). Brains were removed, post-fixed in the same fixative at 4°C for 16 h, and sectioned coronally at 60 µm using a vibratome (DTK-1000N, D.S.K, Japan). Free-floating sections containing the hippocampus were used for immunostaining.

For doublecortin (DCX) staining, sections were blocked for 1 h at room temperature in PBS containing 5% normal serum and 0.3% Triton X-100. Sections were then incubated overnight at 4°C with rabbit anti-DCX primary antibody (Sigma-Aldrich, catalog no. HPA036121, 1:500). On the following day, sections were incubated for 2 h at room temperature with Alexa Fluor 488-conjugated secondary antibody (Abcam, ab150077, 1:1000). Nuclei were counterstained with Hoechst 33342.

For glial marker analysis, sections were incubated with primary antibodies against GFAP and Iba-1 (anti-GFAP, Proteintech, 16825–1-AP, 1:1000; anti-Iba-1, Abcam, ab178846, 1:1500), followed by appropriate fluorescent secondary antibodies. Images were acquired using a confocal laser-scanning microscope (Zeiss, model 700) with 20 × or 40 × objective lenses. For quantitative comparisons within the same experiment, laser power, gain, and exposure settings were kept constant.

### Image analysis

Image analysis was performed using Fiji/ImageJ (NIH). For DCX quantification, regions of interest were defined to include the subgranular zone and the inner border of the granule cell layer in the dentate gyrus. DCX fluorescence intensity was measured within the region of interest. For GFAP and Iba-1 quantification, regions of interest were defined in the somatosensory cortex overlying the dorsal hippocampus at approximately the coronal level of bregma −1.8 mm, and mean fluorescence intensity was measured. For Iba-1, the area of pixels exceeding the predefined threshold was calculated.

An additional exploratory analysis of Iba-1-positive structures was performed using 16-bit, two-dimensional maximum-intensity-projection images. One 512 × 512-pixel image field (150 × 150 µm) from each animal was analyzed (n = 6 mice per condition). Images were processed in ImageJ using a fixed workflow comprising rolling-ball background subtraction (radius, 68.27 pixels), Gaussian smoothing (σ, 0.512 pixels), and local thresholding. Pixels were retained when their smoothed intensity exceeded the local mean (radius, 34.13 pixels) by more than 128 intensity units. Eight-connected components smaller than 3 pixels were removed, and the resulting binary masks were skeletonized in ImageJ. The saved skeleton images were imported into MATLAB and represented as 8-neighbor pixel graphs; redundant diagonal connections were removed when an orthogonal intermediate path was present. Endpoints were defined as degree-1 skeleton pixels, and isolated degree-0 pixels were not counted. The complete field, including boundary-touching components, was retained, and no short-branch pruning was applied.

Processes and endpoints were not assigned to individual cells; this whole-field measure therefore does not quantify branching of individual microglia.

### Data analysis and statistics

Data are presented as mean ± SEM unless otherwise stated. The tail-suspension and contextual-freezing group summaries are reported as mean ± SD. For contextual freezing, each mouse contributed one interval-based percentage score; the 30 scoring intervals were not treated as independent samples. The P28–P56 %age increase in body weight and other multiple-group comparisons at a single time point were analyzed using one-way analysis of variance followed, when appropriate, by Tukey’s multiple-comparison test. Two-group comparisons of behavioral measures, fluorescence intensity, and Iba-1-positive area were performed using unpaired two-tailed Student’s *t*-tests. For the NOR test, the discrimination index of each group was additionally compared with the chance level of zero using a two-tailed one-sample *t*-test. The additional whole-field Iba-1 endpoint counts were compared using a two-sided Welch two-sample *t*-test implemented in Python (SciPy), using one field per mouse (n = 6 mice per condition). The p values for this exploratory endpoint comparison and the additional NOR one-sample tests are unadjusted for multiple comparisons. For the LED two-chamber test, the unpaired two-tailed Student’s *t*-test used each mouse’s total LED-on compartment time as the observation unit. Pooled individual visit durations were displayed descriptively without an inferential group comparison. Statistical analyses of the behavioral, fluorescence-intensity, and positive-area measurements were performed using OriginPro (OriginLab, USA). A value of p < 0.05 was considered statistically significant. Sample sizes are indicated in the corresponding figure legends.

## Results

### Body weight increased in all four dietary groups

In this study, we used a dietary model in which mice received gummies with different mechanical properties. We first examined body weight during the dietary intervention. Representative images of the four diet conditions are shown in Fig. 1A, and the experimental schedule is shown in Fig. 1B. The body weight time course for each group is shown in Fig. 1C. Body weight increased over time from P28 to P56 in all groups, and the principal comparison groups, Soft and Hard, showed broadly similar growth curves. The percentage increase in body weight from P28 to P56 is shown in Fig. 1D (Powder, 49.63 ± 4.65%; Soft, 66.10 ± 6.26%; Hard, 49.60 ± 2.42%; Pellet, 57.54 ± 2.51%). The percentage increase differed significantly among groups (one-way ANOVA, F(3, 20) = 3.38496, p = 0.03832), although no pairwise comparison reached significance after Tukey adjustment (all adjusted p > 0.05). Body weight increased under all four dietary conditions.

### Basal behaviors did not differ significantly across the dietary conditions

We next examined whether the dietary conditions affected basal locomotor activity or basal affect-related behaviors, which could confound the interpretation of subsequent memory-related tasks. The behavioral testing schedule is shown in Fig. 2A. Representative locomotor trajectories in the open field test are shown in Fig. 2B. The Soft and Hard groups showed visually similar exploration patterns, and no significant differences were observed in total distance traveled or time spent in the center zone (Fig. 2C; total distance traveled: Soft vs. Hard, 18.11 ± 1.27 m vs. 16.64 ± 1.71 m, p = 0.52; center time: Soft vs. Hard, 19.77 ± 3.05 s vs. 18.90 ± 4.66 s, p = 0.88). In addition, immobility time in the tail suspension test did not differ significantly among groups (Fig. 2D; mean ± SD: Powder, 85.05 ± 6.06 s; Soft, 84.15 ± 9.01 s; Hard, 86.99 ± 7.64 s; Pellet, 91.21 ± 5.69 s; one-way ANOVA, F(3, 19) = 1.09469, p = 0.37554). These results indicate that, within the scope of the present behavioral assays, no significant differences in these basal behavioral measures were detected under the present dietary conditions.

**Fig. 2:**
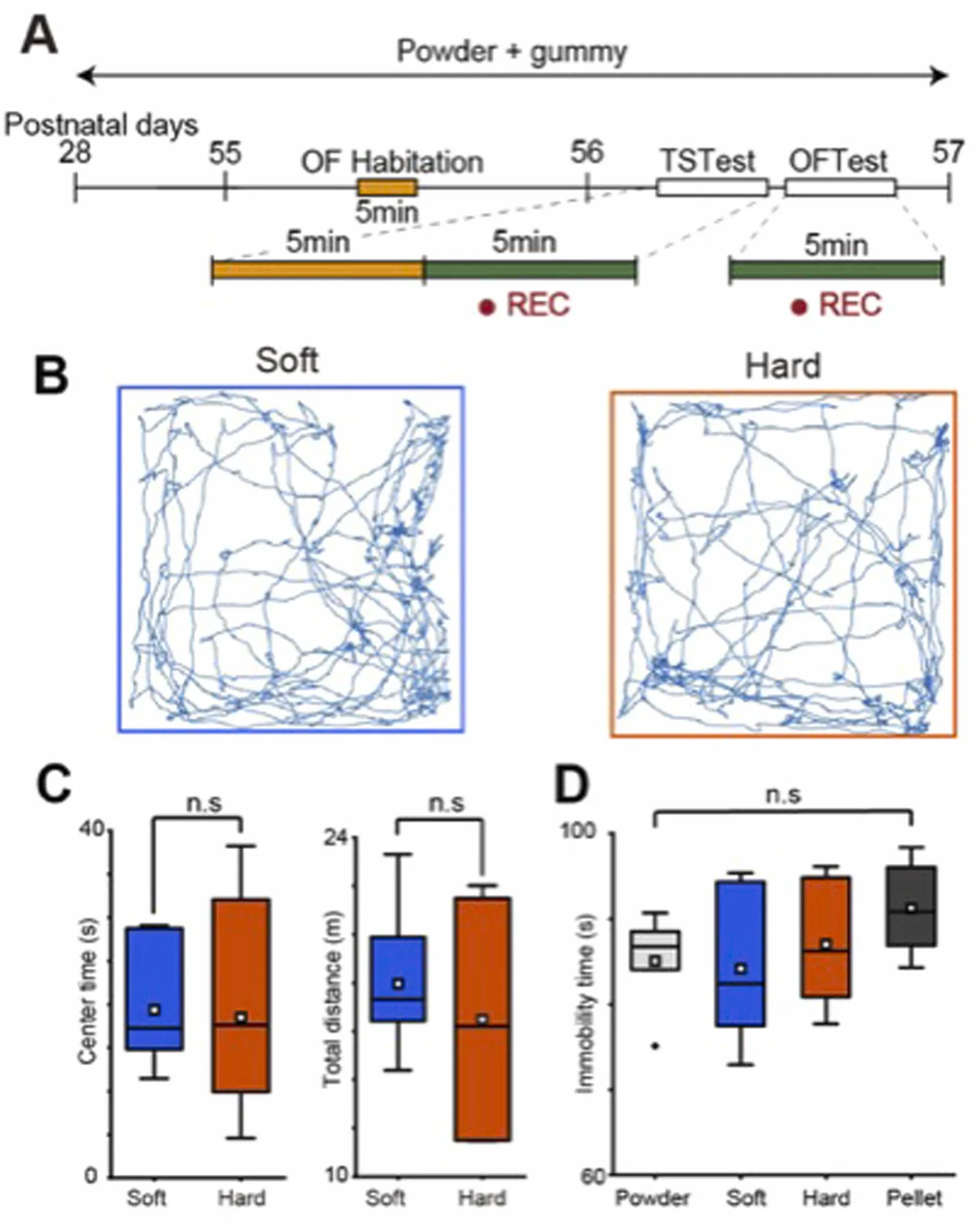
Open field and tail suspension tests. (A) Experimental schedule for the open field test (OFT) and tail suspension test (TST). (B) Representative locomotor trajectories during the OFT in the Soft and Hard groups. (C) Time spent in the center zone (left) and total distance traveled (right) during the OFT. (D) Immobility time in the TST. OFT numerical summaries in the text are mean ± SEM. TST numerical summaries are mean ± SD. For the OFT, Soft: n = 6; Hard: n = 7. For the TST, Powder: n = 5; Soft: n = 6; Hard: n = 6; Pellet: n = 6. n.s., not significant.

### Novel object recognition performance did not differ significantly between the soft and hard groups

Soft-diet feeding or reduced masticatory input has been associated with decreased neurogenesis in the hippocampal dentate gyrus and impaired memory function. Conversely, stronger masticatory loading has been associated with increased survival of newborn cells in the dentate gyrus [6], [7], [8]. Based on these findings, we compared object recognition performance between the Soft and Hard dietary groups. The experimental schedule and objects used for the novel object recognition test are shown in Fig. 3A. Representative locomotor trajectories during the familiarization and test phases are shown in Fig. 3B. The Soft and Hard groups showed broadly similar exploration patterns, and the discrimination index did not differ significantly between the two groups (Fig. 3C; Soft vs. Hard, 0.32 ± 0.076 vs. 0.17 ± 0.18, p = 0.52). In the test phase, the discrimination index was significantly greater than the chance level of zero in the Soft group (t(4) = 4.17, p = 0.01401), whereas it did not differ significantly from zero in the Hard group (t(6) = 0.96, p = 0.37233).

**Fig. 3:**
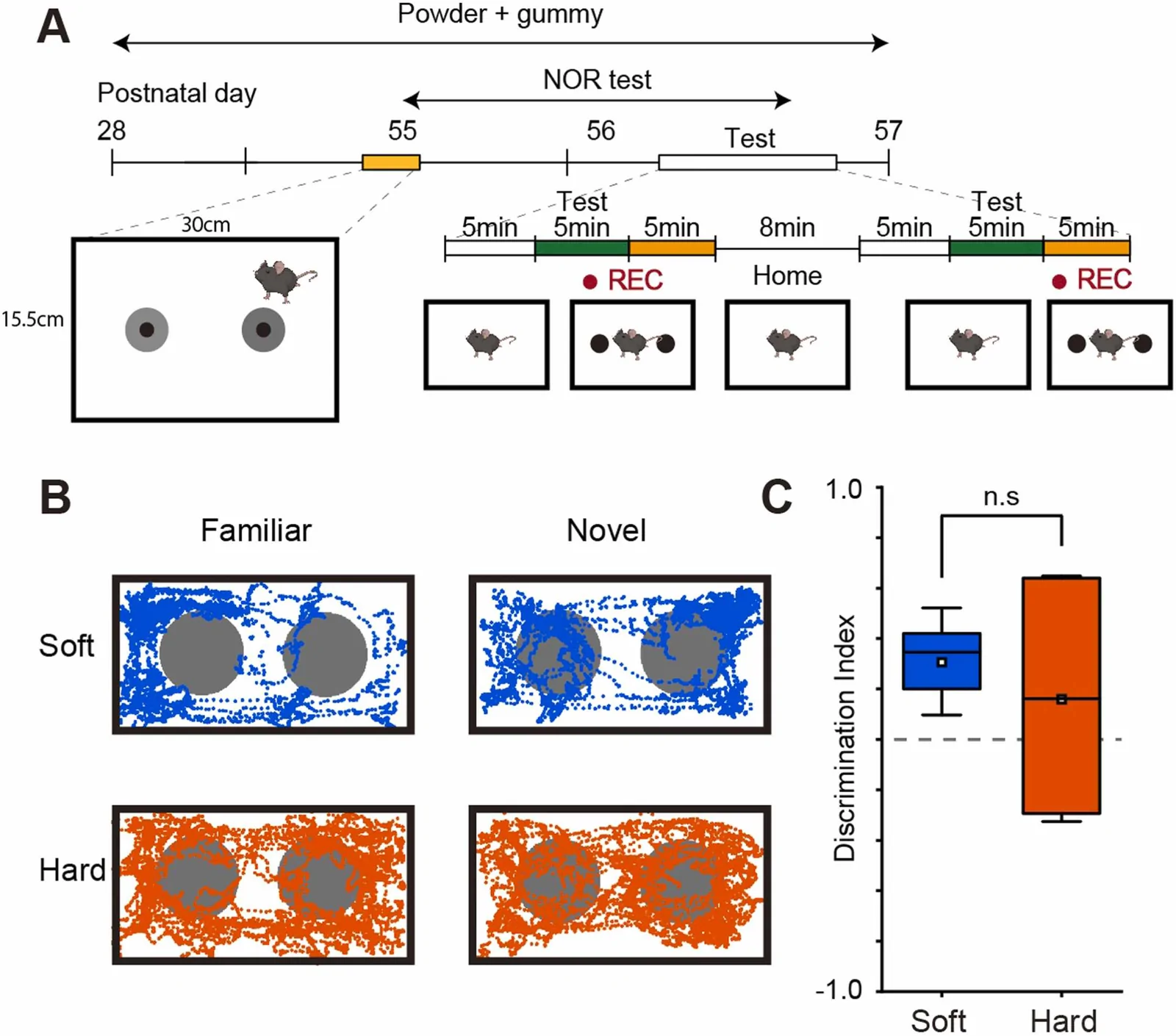
Novel object recognition test. (A) Experimental schedule for the novel object recognition (NOR) test and examples of the objects used. (B) Representative locomotor traces during the familiarization and test phases in the Soft and Hard groups. (C) Discrimination index (DI) in the NOR test. The horizontal dashed line indicates the chance level (DI = 0). Numerical summaries in the text are mean ± SEM. Soft: n = 5; Hard: n = 7. The Soft–Hard comparison used an unpaired two-tailed Student’s *t*-test. Each group’s test-phase DI was additionally compared with zero using a two-tailed one-sample *t*-test. n.s., not significant between groups.

### Contextual freezing differed across dietary conditions; total LED-on compartment time did not differ significantly

The novel object recognition test revealed no significant between-group difference between the Soft and Hard groups. We next examined whether differences associated with the dietary conditions would emerge in associative learning tasks with emotional valence. Chewing under restraint conditions has been reported to attenuate suppression of cell birth in the dentate gyrus, ameliorate hippocampus-dependent spatial memory impairment, and suppress stress-induced hippocampal long-term depression and anxiety-like behaviors [9], [10], [11]. Based on these findings, we performed threat-conditioning tasks to examine behavioral differences associated with the dietary conditions.

The experimental schedule is shown in Fig. 4A. The freezing rate during re-exposure to the conditioning chamber is shown in Fig. 4B. The percentage of freezing-positive intervals differed significantly among groups (one-way ANOVA, F(3, 20) = 14.32646, p = 3.25454 × 10⁻⁵). Tukey’s post hoc test showed higher freezing rates in the Hard and Pellet groups than in the Soft group (mean ± SD: Powder, 40.00 ± 16.33%; Soft, 60.56 ± 9.05%; Hard, 82.78 ± 13.73%; Pellet, 82.78 ± 13.07%; Hard vs. Soft, p = 0.04115; Pellet vs. Soft, p = 0.04115), whereas the Soft–Powder comparison did not reach significance (p = 0.06403). In a separate mixed-sex cohort (Soft, n = 11; Hard, n = 12), we assessed cue-associated behavior using the LED cue. The total time spent in the LED-on compartment did not differ significantly between the Soft and Hard groups (Fig. 4C; Soft vs. Hard: 140.14 ± 9.02 vs. 120.76 ± 8.89 s, p = 0.14129; unpaired two-tailed Student’s *t*-test, t(21) = 1.52858). The distributions of the available individual LED-on compartment visit durations are shown descriptively in Fig. 4D (Soft, 69 visits; Hard, 82 visits).

**Fig. 4:**
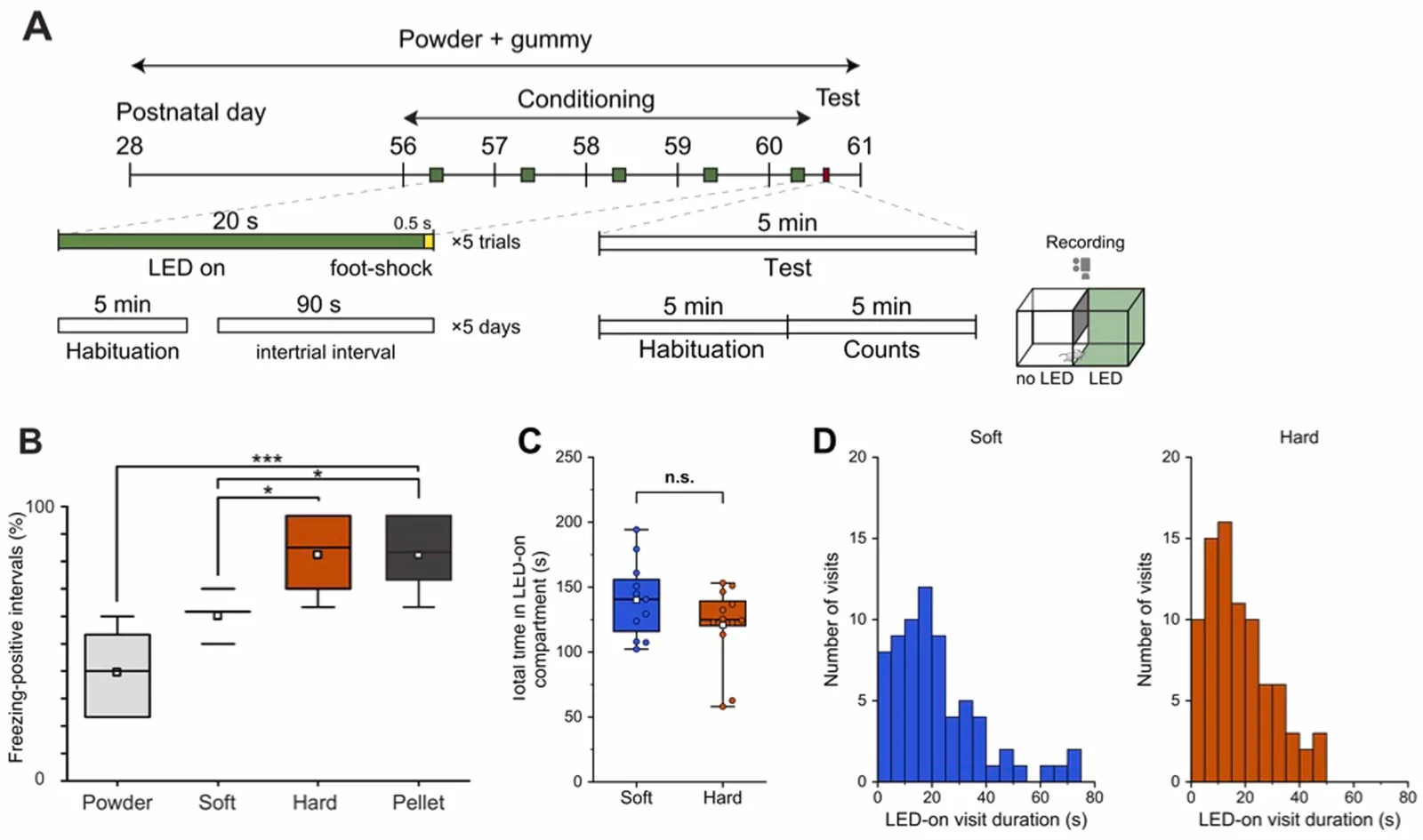
Threat conditioning and cue-associated avoidance tests. (A) Experimental schedules for threat conditioning and behavioral assessments. Contextual fear conditioning and cued conditioning were assessed in separate cohorts. (B) Freezing rate during a 5-min re-exposure to the conditioning chamber at least 30 min after the final foot shock. Mice were returned to their home cages between conditioning and re-exposure. No foot shocks or LED illumination were delivered during the observation period. Freezing rate is expressed as the percentage of 10-s intervals containing at least 2 s of freezing (30 intervals per mouse). Group comparisons used one-way ANOVA followed by Tukey’s multiple-comparison test; brackets indicate pairwise comparisons (*p < 0.05; ***p < 0.001, Tukey-adjusted). (C) Total time spent in the LED-on compartment during the two-chamber test. Colored circles represent individual mice (Soft, n = 11: 6 females and 5 males; Hard, n = 12: 6 females and 6 males). Boxes indicate the interquartile range, horizontal lines indicate medians, white squares indicate group means, and whiskers extend to the minimum and maximum values. The comparison used an unpaired two-tailed Student’s *t*-test; n.s., not significant. (D) Distributions of the available LED-on compartment visit durations in the Soft and Hard conditions. Histograms show the number of visits in common 5-s bins over 0–80 s (Soft, 69 visits; Hard, 82 visits), with identical axis limits for both conditions. These pooled visit-level distributions are presented descriptively; visits were not treated as independent animals for statistical inference. Freezing numerical summaries in the text are mean ± SD. For the freezing test, Powder: n = 6; Soft: n = 6; Hard: n = 6; Pellet: n = 6. Numerical summaries of total LED-on compartment time in the text are mean ± SEM.

### Dentate gyrus DCX labeling and cortical Iba-1-related measures differed between the soft and hard conditions

To examine the histological changes corresponding to the behavioral differences observed in the threat-conditioning task, we analyzed DCX as an immature neuron-related marker in the dentate gyrus and GFAP and Iba-1 as glia-related markers in the cortex.

Representative images of DCX immunofluorescence in the dentate gyrus are shown in Fig. 5A. Quantitative analysis showed that DCX fluorescence intensity was higher in the Hard group than in the Soft group (Fig. 5B; Soft vs. Hard, 859.26 ± 69.92 vs. 1106.36 ± 83.25 arbitrary units, p = 0.046). Representative cortical GFAP images are shown in Fig. 5C. Quantification of cortical GFAP immunoreactivity revealed no significant difference between the Soft and Hard groups (Fig. 5D; Soft vs. Hard, 506.36 ± 45.01 vs. 449.01 ± 30.69, p = 0.32). Representative cortical Iba-1 images are shown in Fig. 5E. Cortical Iba-1 fluorescence intensity was higher in the Hard group than in the Soft group (Fig. 5F; Soft vs. Hard, 370.77 ± 50.17 vs. 590.95 ± 38.40, p = 0.006), and the cortical Iba-1-positive area was also higher in the Hard group (Fig. 5G; Soft vs. Hard, 8703.33 ± 2419.16 vs. 18071.33 ± 2588.79, p = 0.025). These results indicate that the Hard condition was associated with increased DCX immunoreactivity in the dentate gyrus and increased cortical Iba-1 labeling, whereas cortical GFAP immunoreactivity did not differ significantly under the present conditions.

**Fig. 5:**
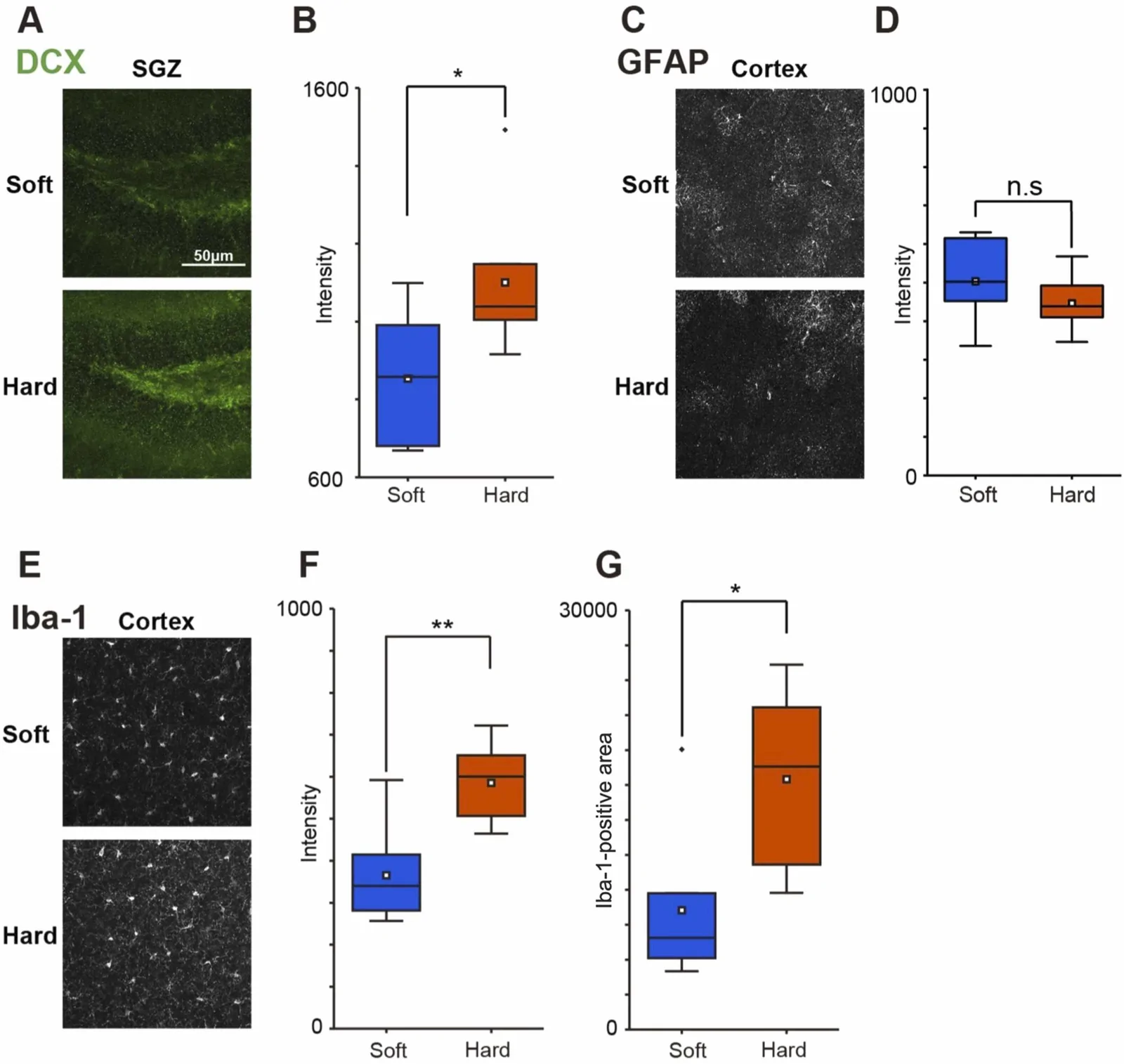
Dentate gyrus DCX labeling and cortical GFAP/Iba-1 immunostaining under different diet conditions. (A) Representative images of doublecortin (DCX) immunofluorescence in the dentate gyrus (DG) of the Soft and Hard groups. Hoechst staining was used for nuclear counterstaining. (B) Quantification of DCX fluorescence intensity in the DG. (C) Representative images of glial fibrillary acidic protein (GFAP) immunostaining in the cortex. (D) Quantification of GFAP immunoreactivity in the cortex. (E) Representative images of ionized calcium-binding adaptor molecule-1 (Iba-1) immunostaining in the cortex. (F) Quantification of Iba-1 fluorescence intensity in the cortex. (G) Quantification of Iba-1-positive area in the cortex. Numerical summaries in the text are mean ± SEM. n = 6 mice per group. Scale bars, 50 μm. n.s., not significant; *p < 0.05; **p < 0.01.

To further characterize the increased cortical Iba-1 labeling, we performed an exploratory whole-field skeleton analysis. Example maximum-intensity projections, selected skeleton overlays, and illustrative endpoint markings are shown in Fig. 6A. Whole-field skeleton endpoint counts were higher in the Hard condition than in the Soft condition (Fig. 6B; Soft vs. Hard: 3293.00 ± 157.14 vs. 4124.33 ± 53.46 endpoints/field, p = 0.002273; two-sided Welch *t*-test, t(6.14) = 5.01; n = 6 mice per group). One field from each animal was analyzed.

**Fig. 6:**
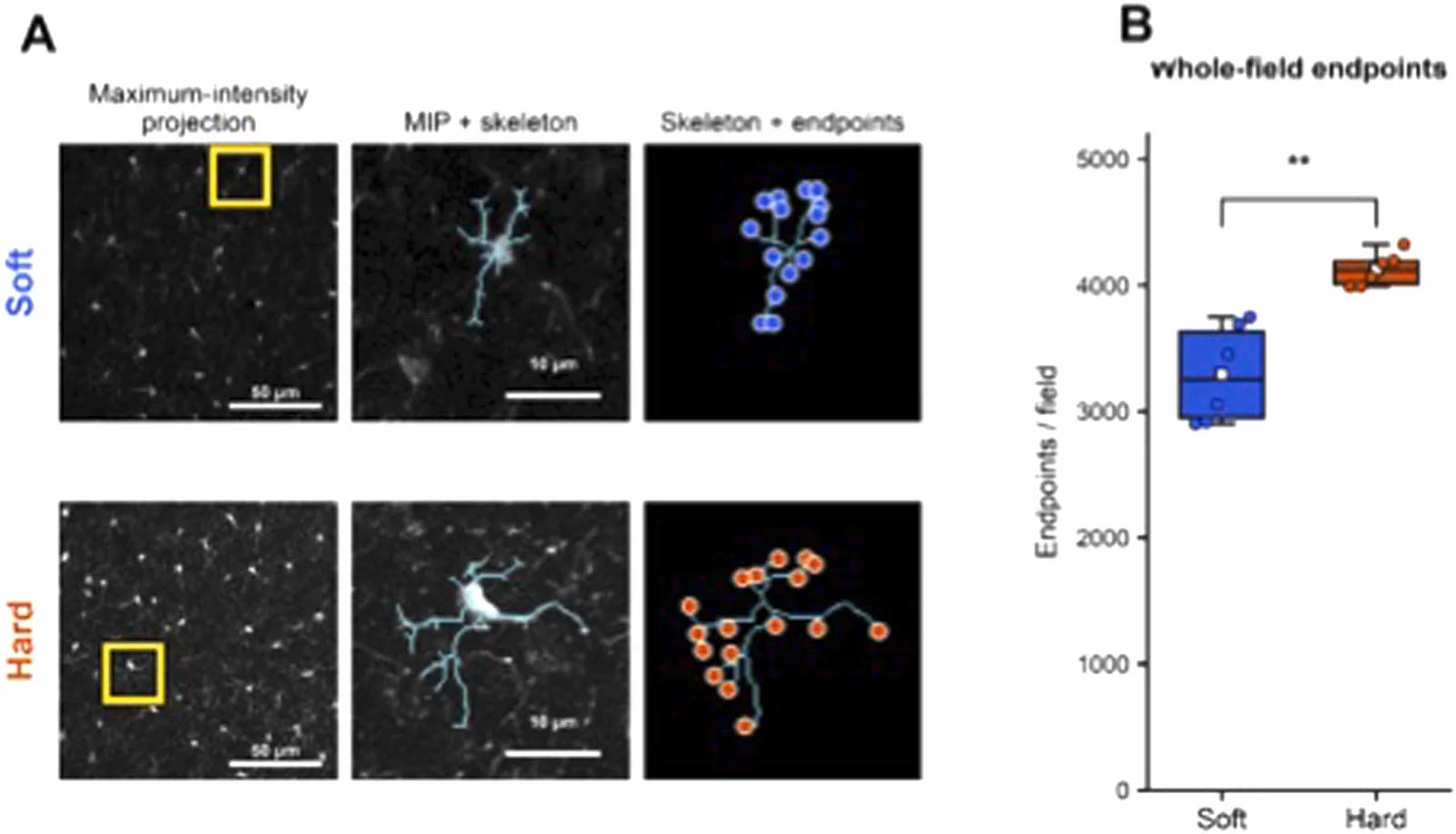
Field-level analysis of cortical Iba-1-positive structures in the Soft and Hard conditions. (A) Example maximum-intensity projections (MIPs) of cortical Iba-1 immunofluorescence in the Soft (upper row) and Hard (lower row) conditions. Yellow boxes indicate the regions shown at higher magnification in the middle and right columns. The middle column shows enlarged MIPs overlaid with selected skeletonized structures (cyan), and the right column shows these structures with their endpoints marked by blue (Soft) or orange (Hard) circles. The selected structures illustrate endpoint identification; the quantitative measurements in B were obtained from complete image fields, not these displayed subsets. Scale bars, 50 μm (full-field images) and 10 μm (enlarged images). (B) Total number of skeleton endpoints per image field. One 512 × 512-pixel field (150 × 150 µm) was analyzed per mouse (n = 6 mice per group). Endpoints were not assigned to individual cells; this measure does not quantify branching of individual microglia. For panel B, colored circles represent measurements from individual mice, and white squares indicate group means. Boxes indicate the interquartile range, horizontal lines indicate medians, and whiskers indicate the minimum and maximum values. The comparison used a two-sided Welch *t*-test; **p < 0.01 (unadjusted). Numerical summaries in the text are mean ± SEM.

## Discussion

In this study, we used a dietary model based on gummies with different mechanical properties, and examined behavior, dentate gyrus DCX labeling, and cortical glia-related markers. In the principal comparison between the Soft and Hard groups, body weight increased (Fig. 1C, D), and no significant differences were detected in basal locomotor activity, center-zone time, or tail-suspension immobility (Fig. 2C, D). Although non-emotional object recognition did not differ significantly between these groups, the discrimination index was significantly above zero in Soft but not Hard (Fig. 3C). Freezing scores during re-exposure to the conditioning chamber differed according to the diet condition (Fig. 4B), whereas total LED-on compartment time did not differ significantly between Soft and Hard in a separate mixed-sex cohort (Fig. 4C). Histologically, the Hard group showed higher DCX immunoreactivity in the dentate gyrus and higher cortical Iba-1 fluorescence intensity and positive area, whereas cortical GFAP immunoreactivity did not differ significantly (Fig. 5B, D, F, G). Exploratory image analysis additionally showed higher whole-field endpoint counts in Hard (Fig. 6B). These findings suggest that differences in gummy mechanical properties are associated with threat-related behavior and neuroglial markers.

The conceptual motivation for considering clenching in the present study was that it is a high-load oral motor act distinct from rhythmic chewing. Chewing and isometric biting/clenching differ in jaw-muscle activation patterns and jaw gape even when comparable levels of bite force are produced [1]. Voluntary teeth clenching has also been shown to facilitate limb reflex excitability and motor system excitability [3], [4], [5]. Whether the observed freezing difference reflects clenching-related oral motor load remains to be determined. The Powder and Pellet groups served as reference conditions rather than as validation of a clenching-specific manipulation. The parallel direction of the Soft–Hard and Powder–Pellet freezing patterns is also compatible with broader effects of dietary hardness or mastication.

Non-emotional object recognition did not clearly differ between the Soft and Hard groups (Fig. 3C). At first glance, this may appear inconsistent with previous studies showing that soft-diet feeding or reduced mastication is associated with reduced hippocampal neurogenesis and impaired memory function [6], [7], [12]. However, most of these models substantially alter mastication itself, oral sensory input, and related peripheral conditions. In the present study, the discrimination index was significantly above the chance level of zero in the Soft group, whereas it did not differ significantly from zero in the Hard group. The result in the Soft group is consistent with object recognition memory under the present test conditions. However, the difference in within-group significance does not itself establish impaired recognition in the Hard group or a significant difference between the two dietary conditions. The nonsignificant between-group comparison also does not establish equivalent recognition performance.

In contrast, freezing scores during re-exposure to the conditioning chamber differed among the dietary conditions. The percentage of freezing-positive intervals was higher in the Hard and Pellet groups than in the Soft group (Fig. 4B). In the separate mixed-sex cue-associated cohort, total LED-on compartment time did not differ significantly between the Soft and Hard groups (Fig. 4C). This result does not provide independent support for enhanced cue-associated avoidance. The freezing findings indicate that the dietary conditions were associated with differences in behavioral expression in a threat-associated setting. Greater freezing can be consistent with stronger threat memory, greater generalization, poorer discrimination, or altered expression of defensive behavior; the present experiment does not distinguish these explanations. In particular, an interpretation based on greater generalization or poorer discrimination would run counter to an account based on improved pattern separation. It should be noted, however, that the present task did not directly assess classical contextual fear discrimination. Therefore, the present data do not allow us to conclude that pattern separation was improved. Previous studies have shown that suppression of adult hippocampal neurogenesis impairs contextual fear conditioning, deletion of NMDA receptors in the dentate gyrus disrupts discrimination between similar contexts, and increasing adult hippocampal neurogenesis improves pattern separation [14], [15], [16]. These studies informed the rationale for examining dentate gyrus markers but are not used as supportive evidence for improved pattern separation or for the behavioral differences in the present study.

The histological findings provide further evidence of differences in neuroglial markers between the Soft and Hard conditions. The Hard group showed higher DCX immunoreactivity in the dentate gyrus than the Soft group (Fig. 5A, B), consistent with a previous report showing that stronger masticatory loading increased the survival of newborn cells in the dentate gyrus [8]. The present DCX measurement reflects fluorescence intensity and does not directly quantify neurogenesis or newborn-cell survival. In the cortex, Iba-1 fluorescence intensity and Iba-1-positive area were higher in the Hard group (Fig. 5E–G). Exploratory image analysis additionally showed higher whole-field skeleton endpoint counts in Hard (Fig. 6B). In view of the somatosensory cortical rationale described above, the cortical Iba-1 findings may represent an additional cellular correlate of the dietary conditions outside the dentate gyrus. The additional endpoint measurement characterizes skeletonized Iba-1-positive structures within the sampled fields. Because processes were not assigned to individual cells, the endpoint count does not establish increased ramification of individual microglia. However, Iba-1 intensity, positive area, and skeleton endpoint measurements alone cannot define microglial activation states. In contrast, cortical GFAP immunoreactivity did not differ significantly between the Soft and Hard groups (Fig. 5C, D). More detailed single-cell morphological analyses, such as soma size, process length, branching complexity, and Sholl analysis, would be required to characterize microglial morphology; independent functional measurements would be needed to establish an anti-inflammatory or surveillant state.

The mechanism linking the behavioral and histological differences observed in this study remains speculative. However, a possible pathway can be considered. A recent study in healthy adults reported that external trigeminal nerve stimulation induced greater pupil dilation than control stimulation, suggesting involvement of the locus coeruleus-norepinephrine system [20]. This finding provides a rationale for considering trigeminal and arousal-related modulatory pathways. It is therefore possible that differences in oral sensory or motor input associated with the different gummy mechanical properties affected the dentate gyrus microenvironment or the formation and expression of threat-related behavior through trigeminal pathways. However, because the present study did not directly measure jaw-muscle electromyography, bite force, locus coeruleus activity, or brain monoamine dynamics, this pathway should be regarded as a candidate mechanism for future investigation rather than as a conclusion of the present study.

Taken together, the present study provides an experimental step toward examining how differences in gummy mechanical properties are associated with behavioral and neuroglial measures. Under the present conditions, the between-group difference in non-emotional object recognition was not significant, whereas differences were observed in threat-related behavior, dentate gyrus DCX labeling, and cortical glia-related markers. In this sense, the findings motivate further investigation of clenching and other oral motor components, although the present study did not directly isolate clenching from general mastication. Considering that occlusal force has been associated with cognitive function in humans [13], the present findings highlight the need to decompose oral and masticatory behaviors into more specific motor components when considering their relationship with brain function.

This study has several limitations. First, the present study compared gummies with different mechanical properties, but did not directly measure bite force or jaw-muscle activity. Therefore, the extent to which the difference in maximum compression load of the gummies translated into differences in oral motor or clenching load remains to be determined. Actual gummy consumption was not quantitatively recorded, and the supplied amount does not establish equivalent intake between the Soft and Hard groups. Differences in gelatin concentration, palatability, or consumption remain possible contributors. Comparisons between the gummy-supplemented and reference diets additionally differ in gummy constituents, including sugar, and potentially in palatability. Second, the analyses of DCX, GFAP, and Iba-1 were correlational, and the present study does not demonstrate that these histological changes causally mediated the differences in threat-related behavior. In addition, the whole-field skeleton endpoint analysis provides a quantitative field-level measure but does not quantify the morphology of individual microglia. Further single-cell morphological and functional analyses would be required to define microglial structure and state more precisely. The LED visit-duration histograms are descriptive because visit-to-animal linkage was unavailable; moreover, 69 Soft visit durations were retained although the archived visit-count table totals 70. Animal-level inference uses the separately recorded total LED-on times. Third, the main experimental cohorts used young adult male C57BL/6 J mice, whereas the separate LED cue-associated avoidance cohort included both sexes. The reported LED Soft–Hard comparison pooled sexes and the study was not designed to test sex-dependent effects; potential sex effects and aging therefore remain unexplored. Fourth, the assessment of affect-related memory was limited to a threat-conditioning paradigm, and the generalizability of the findings to other emotional domains, such as reward-related or social memory, remains unclear. Because the main behavioral tests were performed in a fixed order in the same mice, potential effects of preceding tests on subsequent behavioral measurements cannot be excluded. Future studies combining direct measurements of jaw-muscle activity and bite force with manipulations of dentate gyrus plasticity or glial function, quantitative microglial morphology, and models incorporating sex and aging will be necessary to further test the potential contribution of clenching-related oral motor load to brain plasticity.

## CRediT authorship contribution statement

**Haruka Sasaki:** Writing – review & editing, Writing – original draft, Visualization, Methodology, Investigation, Formal analysis, Conceptualization. **Minori Tajima:** Visualization, Methodology, Formal analysis, Data curation, Conceptualization. **Ririka Morimoto:** Formal analysis, Data curation. **Chihiro Kobayashi:** Formal analysis, Data curation. **Yumi Miki:** Formal analysis, Data curation. **Yuka Hirota:** Formal analysis, Data curation. **Hiromu Monai:** Writing – review & editing, Writing – original draft, Project administration, Methodology, Investigation, Funding acquisition, Formal analysis, Conceptualization.

## Ethics declaration

This study was conducted in accordance with the ARRIVE (Animal Research: Reporting of In Vivo Experiments) guidelines. This study was approved by the Institutional Animal Care and Use Committee of Ochanomizu University. (Approval Nos. 23007, 24017, and 25007)

## Declaration of Competing Interest

The authors declare that they have no known competing financial interests or personal relationships that could have appeared to influence the work reported in this paper.

## Data Availability

The data that support the findings of this study are available from the corresponding author upon reasonable request.

## References

[1] Pröschel P.A., Jamal T., Morneburg T.R. (2008). Motor control of jaw muscles in chewing and in isometric biting with graded narrowing of jaw gape. J Oral Rehabil.

[2] Sasaki Y., Ueno T., Taniguchi H., Ohyama T. (1998). Effect of teeth clenching on isometric and isokinetic strength of ankle plantar flexion. J Med Dent Sci.

[3] Miyahara T., Hagiya N., Ohyama T., Nakamura Y. (1996). Modulation of human soleus H reflex in association with voluntary clenching of the teeth. J Neurophysiol.

[4] Takada Y., Miyahara T., Tanaka T. (2000). Modulation of H reflex of pretibial muscles and reciprocal Ia inhibition of soleus muscle during voluntary teeth clenching in humans. J Neurophysiol.

[5] Boroojerdi B., Battaglia F., Muellbacher W., Cohen L.G. (2000). Voluntary teeth clenching facilitates human motor system excitability. Clin Neurophysiol.

[6] Yamamoto T., Hirayama A., Hosoe N. (2009). Soft-diet feeding inhibits adult neurogenesis in hippocampus of mice. Bull Tokyo Dent Coll.

[7] Fukushima-Nakayama Y., Ono T., Ono T. (2017). Reduced mastication impairs memory function. J Dent Res.

[8] Akazawa Y., Kitamura T., Fujihara Y. (2013). Forced mastication increases survival of adult neural stem cells in the hippocampal dentate gyrus. Int J Mol Med.

[9] Kubo K. ya, Sasaguri K., Ono Y. (2009). Chewing under restraint stress inhibits the stress-induced suppression of cell birth in the dentate gyrus of aged SAMP8 mice. Neurosci Lett.

[10] Miyake S., Yoshikawa G., Yamada K. (2012). Chewing ameliorates stress-induced suppression of spatial memory by increasing glucocorticoid receptor expression in the hippocampus. Brain Res.

[11] Ono Y., Koizumi S., Onozuka M. (2015). Chewing prevents stress-induced hippocampal LTD formation and anxiety-related behaviors: a possible role of the dopaminergic system. Biomed Res Int.

[12] Piancino M.G., Tortarolo A., Polimeni A. (2020). Altered mastication adversely impacts morpho-functional features of the hippocampus: a systematic review on animal studies in three different experimental conditions involving the masticatory function. PLoS One.

[13] Ikebe K., Gondo Y., Kamide K. (2018). Occlusal force is correlated with cognitive function directly as well as indirectly via food intake in community-dwelling older Japanese: from the SONIC study. PLoS One.

[14] Saxe M.D., Battaglia F., Wang J.W. (2006). Ablation of hippocampal neurogenesis impairs contextual fear conditioning and synaptic plasticity in the dentate gyrus. Proc Natl Acad Sci USA.

[15] McHugh T.J., Jones M.W., Quinn J.J. (2007). Dentate gyrus NMDA receptors mediate rapid pattern separation in the hippocampal network. Science.

[16] Sahay A., Scobie K.N., Hill A.S. (2011). Increasing adult hippocampal neurogenesis is sufficient to improve pattern separation. Nature.

[17] Tamura Y., Shibukawa Y., Shintani M. (2008). Oral structure representation in human somatosensory cortex. Neuroimage.

[18] Clemens A.M., Fernandez Delgado Y., Mehlman M.L. (2018). Multisensory and motor representations in rat oral somatosensory cortex. Sci Rep.

[19] Mathis A., Mamidanna P., Cury K.M. (2018). DeepLabCut: markerless pose estimation of user-defined body parts with deep learning. Nat Neurosci.

[20] Seminck N., Khatoun A., Kerstens S. (2024). External stimulation of the trigeminal nerve causes pupil dilation in healthy volunteers, suggesting locus coeruleus modulation. Brain Stimul.

